# Opioid Overdose Patients in Central Missouri, United States, Have High Rates of Hepatitis C Infection and Limited Testing History

**DOI:** 10.7759/cureus.67140

**Published:** 2024-08-18

**Authors:** John A Swift, Julie Stilley

**Affiliations:** 1 Infectious Diseases, University of Missouri School of Medicine, Columbia, USA; 2 Emergency Medicine, University of Missouri School of Medicine, Columbia, USA

**Keywords:** hepatitis c screening, emergency department, opioid overdoses, hiv testing, hepatitis c (hcv) infection

## Abstract

Background: Cases of newly identified hepatitis C virus (HCV) infection increased 3.8-fold between 2010 and 2017 due to increasing injection drug use. Furthermore, multiple HIV outbreaks have been attributed to injection drug use. This retrospective cohort study assessed the prevalence of and testing history for HIV and HCV among opioid overdose patients in the emergency department.

Methods: Each encounter including an opioid overdose at three emergency departments between January 2021 and May 2022 was reviewed. Emergency department note, most recent primary care note, and laboratory results from January 2000 to May 2022 were reviewed for the history of HIV and HCV testing. Fisher’s exact test was used to identify associations of HIV and HCV status with age or gender.

Results: There were 134 encounters for 120 patients. A total of 72 were male and 48 were female. A total of 48 had a history of HCV testing. A total of 54 had a history of HIV testing. A total of 20 tested positive for HCV antibodies. One tested positive for HIV. Eight had detectable HCV viral loads, six had undetectable HCV viral loads, and six had no quantitative testing. One had a detectable HIV viral load. A total of 16.7% of both males and females had a history of a positive HCV test. Females were more likely to have ever received an HCV test compared to males (p=0.013, odds ratio (OR)=.68 (confidence interval (CI): 1.293-5.836)). Patients aged 55-64 were more likely to test positive than any other age group (p=0.018, OR=3.889 (CI: 1.391-11.81)), and were the least likely to be untested (p=0.037, OR=0.1905 (CI: 0.03914-0.9334)).

Conclusion: There is a substantial burden of HCV among opioid overdose patients in central Missouri, United States, emergency departments, particularly among male patients and those aged 55-64. Universal HCV screening for individuals being observed following an overdose could detect many undiagnosed HCV infections.

## Introduction

In the United States, hepatitis C virus (HCV) is the most common blood-borne pathogen [1] and cases of newly acquired HCV infection increased by approximately 3.8-fold between 2010 and 2017 [2]. A suspected driver for the recent increase in HCV infections is an increase in injection drug use, as well as increased surveillance for the infection [2]. Additionally, despite only accounting for 8% of all new reported HIV infections in 2021 [3], multiple large local outbreaks of HIV in Europe, North America, and Israel since 2010 have occurred among people who inject drugs [4]. While injection drug use has long been recognized as a risk factor for acquiring HCV, recent research has identified elevated HCV antibody reactivity rates amongst patients receiving oral prescription opioids and recommended that their use should be considered a risk factor for HCV infection given the risk of progression from oral to intravenous opioid use [5].

Emergency departments occupy a unique position in the identification of infections such as HIV and HCV. Underserved populations that are disproportionately impacted by HCV, including minorities, the uninsured, and Medicaid recipients, are more likely to utilize emergency departments [6]. Additionally, emergency departments stand to benefit from earlier detection and treatment of these infections, as total annual costs of all HCV-associated visits increased by 192% between 2006 and 2014 [7]. In response, multiple hospitals across both urban and non-urban settings have investigated the prevalence of HCV among all emergency department patients by implementing non-targeted opt-out screening programs. These programs have identified a high prevalence of HCV among emergency department patients, including a large number of cases that were previously undiagnosed [6,8].

Today, in the United States, the University of Missouri Hospital System emergency departments do not have a routine screening program to test any patients for HIV or HCV. Since patients using opioids may have an elevated risk of acquiring HIV and HCV, there is a concern that many patients who present to the emergency department following an opioid overdose may be asymptomatically infected with HIV or HCV and be undiagnosed. Many patients may be from disadvantaged backgrounds and have limited access to healthcare services, making emergency departments well-situated to detect these infections and link patients to care. This retrospective cohort study evaluated HIV and HCV prevalence and testing history among patients presenting to the emergency department following an overdose. The objective of this study was to evaluate the frequency of HIV and HCV testing among patients presenting to the emergency department following an opioid overdose and whether this population had increased rates of HIV and HCV, in order to establish whether routine screening for these infections would be beneficial in identifying previously unidentified HIV and HCV infections.

This article was previously presented as a poster at the 17th World Congress on Public Health in 2023 and the American Medical Association's 2023 annual meeting.

## Materials and methods

Electronic health record data were used for this analysis. Every encounter at three emergency departments in one health system between January 1, 2021, and May 31, 2022, that included a diagnosis code of “poisoning by other opioids, accidental (unintentional)” was included in this analysis. These hospitals constitute the largest hospital system in central Missouri and care for a large proportion of the overdose patients in Columbia and Jefferson City, as well as the surrounding communities of central Missouri. Institutional Review Board approval was obtained before beginning the study.

For each overdose patient, the emergency department note for their visit related to their opioid overdose, emergency medical services transport note, most recent primary care note, and laboratory results dating back to January 1, 2000, were reviewed for any history of HIV and HCV testing. For patients who had a history of a positive HIV or HCV test, the laboratory results dating to January 1, 2000, were also used to assess whether they had a detectable viral load, had no history of quantitative testing, or were virologically suppressed in the setting of HIV infection. Additionally, the emergency department note and emergency services transfer note were used to categorize the route of opioid administration used by the patients. If a patient denied using drugs, no information regarding the route of drug administration was available, or if any sources conflicted in their report on the route of drug use, the encounter was classified as “unclear or unknown.” Fisher’s exact test was used to assess if emergency department visits, testing patterns, or positivity rates differed based on age or gender. Differences between groups were considered significant if the calculated p-value was <0.05. Each patient’s gender was determined using the patient’s stated gender identity as recorded in their electronic health record. The age of each patient corresponds to their age at presentation to the emergency department.

## Results

A total of 134 encounters were identified. Seven visits were determined to be duplicates and were removed, leaving 127 encounters for 121 unique patients. One patient was found to have conflicting information in the clinical encounter notes in their health record regarding their HIV and HCV status, which could not be rectified using laboratory results, so they were excluded from the analysis, leaving 126 encounters for 120 patients.

**Figure 1:**
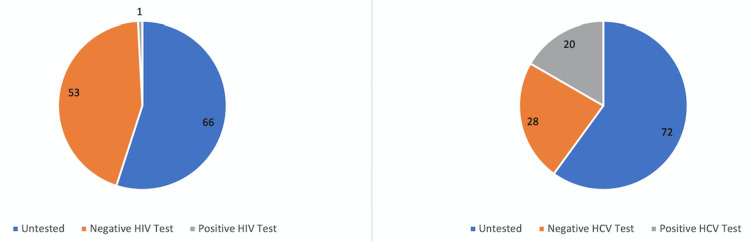
HIV and HCV testing history. The number of patients having received a positive test, negative test, and having never received a test for HIV and HCV is shown. A total of 120 patients were seen in the University of Missouri and Capital Region Medical Center emergency departments in the United States following an opioid overdose between January 1, 2021, and May 31, 2022. A total of 54 patients (45%) had a documented history of an HIV test, with one patient having a positive test (1.9% of those tested, 0.8% of the total population). A total of 48 (40%) had a prior HCV test, with 20 having a positive test (41.7% of the tested, 16.7% of the total population). HCV: hepatitis C virus

**Figure 2:**
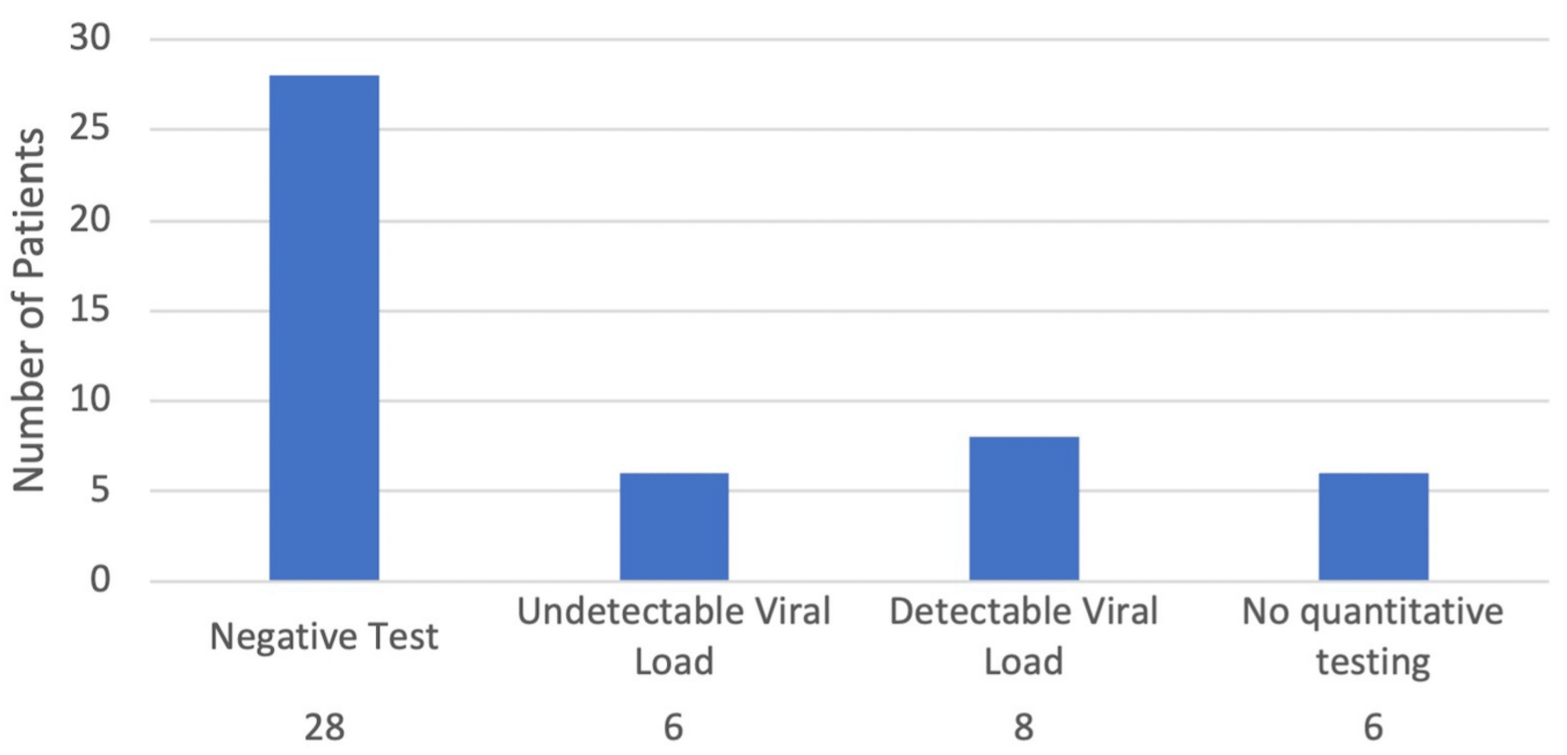
HCV viral load status: the number of patients who had received an HCV test and the results of their viral load testing. A total of 48 out of 120 (40%) patients had received an HCV test. A total of 28 (70% of the tested, 23.3% of the total population) had received a negative test. Among the 20 patients with a positive test, six (30%) had undetectable viral loads, eight (40%) had detectable viral loads, and six (30%) had no documented history of quantitative testing.

The age and sex demographics of the 120 patients and their relation to HCV testing history are represented in Table 1. Table 2 shows the route of opioid administration for the 126 emergency department visits as well as the relation of the route of opioid administration to the HCV testing status for the 120 patients. The routes of drug administration used for each emergency department visit, as well as the HCV testing status of the patients are shown. Figure 1 shows the prevalence of HIV and HCV testing and testing results among this sample. A total of 54 patients (45%) had a documented history of an HIV test, while 48 (40%) had a documented HCV test history. One patient had a positive HIV test (1.9% of patients tested for HIV, 0.8% of the total population), while 20 had a positive HCV test (41.7% of patients tested for HCV, 16.7% of the total population). Figure 2 demonstrates the HCV viral load status in the study population. Six patients (30% of individuals with viral load testing, 5% of the total population) had a quantitative test that showed an undetectable viral load, eight (40% of individuals with viral load testing, 6.67% of the total population) had quantitative tests that revealed a detectable viral load, and six (30% of individuals with viral load testing, 5% of the total population) had no quantitative testing history. Additionally, one patient with a positive HIV test was found to have a detectable viral load.

**Table 1:** Emergency department patient demographics. Sex and age characteristics of 120 emergency department visits following an opioid overdose and their relation to hepatitis C testing history. HCV: hepatitis C virus

	Total		HCV +		HCV -		No HCV testing history	
	n	%	n	%	n	%	n	%
Total ED Patients	120	100	20	16.7	28	23.3	72	60
Female	48	40	8	16.7	18	37.5	22	45.8
Male	72	60	12	16.7	10	13.9	50	69.4
<18	5	4.2	0	0	2	40	3	60
18-24	20	16.7	0	0	5	25	15	75
25-34	45	37.5	5	11.1	10	22.2	30	66.7
35-44	26	21.7	6	23.1	4	15.4	16	61.5
45-54	11	9.2	2	18.2	5	45.5	4	36.7
55-64	9	7.5	7	77.8	0	0	2	22.2
65+	3	2.5	0	0	2	66.7	1	33.3
Unknown	1	0.8	0	0	0	0	1	100

**Table 2:** Route of opioid administration leading to overdose and the relation to HCV testing status. The number and percentage of 126 emergency department visits related to routes of drug administration are given. The number and percentage of 120 emergency department patients organized by HCV status in relation to the route of opioid administration are also given. *One patient with no HCV testing history had multiple ED visits following different routes of drug administration. Thus, they are included in both the “unclear or unknown” and “snorted” categories. HCV: hepatitis C virus

	Total		HCV +		HCV -		No HCV Testing History	
	n	%	n	%	n	%	n	%
Intravenous	15	11.9	4	26.7	1	6.7	10	66.7
Snorted	35	27.8	6	18.2	9	27.3	18*	54.5
Smoked	5	4	0	0	2	50	2	50
Oral	21	16.7	0	0	6	30	14	70
Unclear or unknown	50	39.7	10	20.4	10	20.4	29*	59.2

**Figure 3:**
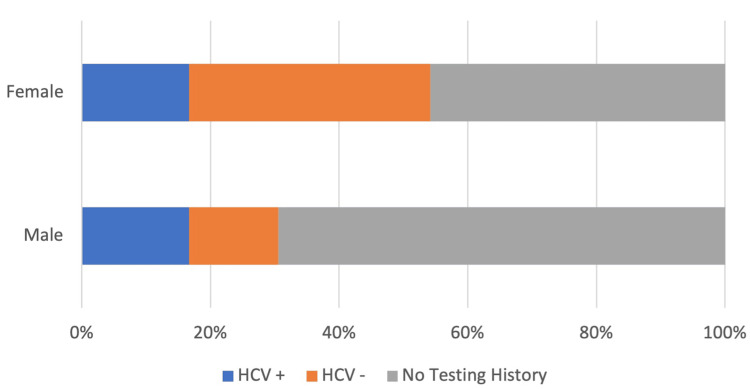
HCV testing differences between genders. Percent of each gender having received a negative HCV test, positive HCV test, and having no HCV testing history. A total of 16.7% (n=12) of males and 16.7% (n=8) of females in the study population had a history of a positive HCV test. A total of 13.9% (10) of males and 37.5% (n=18) of females had received a negative HCV test. Females were more likely to have ever received an HCV test (p=0.013, OR=2.68 (CI: 1.293 to 5.836)), with 45.8% (n=22) of women having no history of HCV testing compared to 69.4% (n=50) of males. HCV: hepatitis C virus

Because there was only one positive HIV test in the sample, demographic analysis was only done with reference to HCV status. Of the 120 patients in the analysis, 72 (60%) were male and 48 (40%) were female, making females significantly less likely to present to the emergency department due to an overdose compared to males (p=0.035). Figure 3 shows the differences in HCV testing patterns between males and females. A total of 16.7% (n=12) of males and 16.7% (n=8) of females had received a positive HCV test. Females, however, were more likely to have ever received an HCV test (p=0.013, OR=2.68 (CI: 1.293 to 5.836)), with 45.8% (n=22) of females having no history of HCV testing compared to 69.4% (n=50) of males.

**Figure 4:**
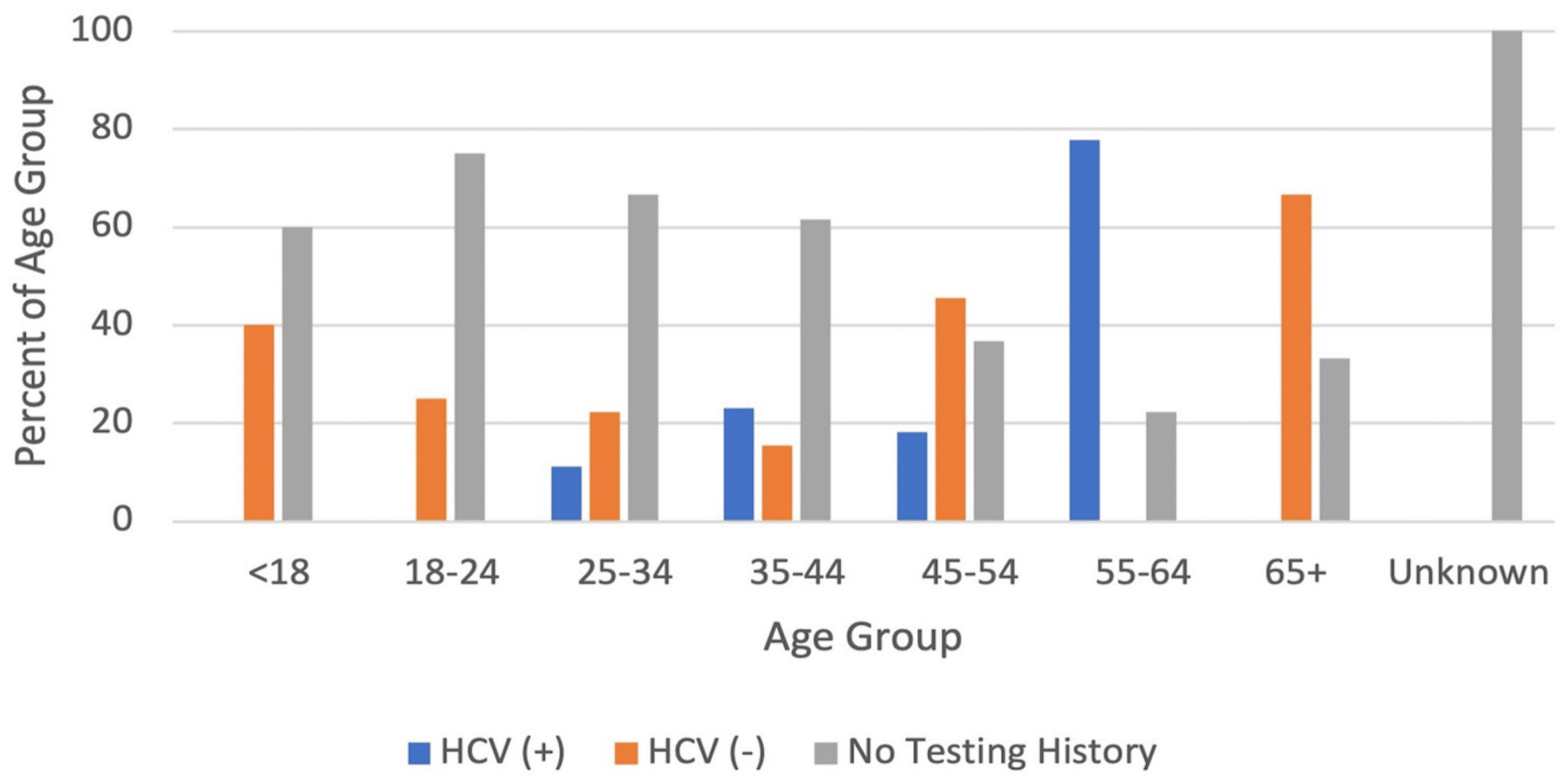
HCV testing patterns based on age. Percent of each age group having received a negative HCV test, positive HCV test, and having no HCV testing history. Patients aged 55-64 were less likely to be untested for HCV compared to the remainder of the study population (p=0.037, OR: 0.1905 (CI: 0.03914 to 0.9334)). They were also more likely to test positive for HCV than any other age group (p=0.018, OR: 3.889 (CI: 1.391 to 11.81)). HCV: hepatitis C virus

The most common age range for patients was 25-34 years with 45 patients, representing 37.5% (n=54) of the total study population. The median age of the study population was 33 years. Figure 4 shows differences in HCV testing results by age. Patients aged 55-64 years were less likely to be untested compared to the rest of the population (p=0.037, OR=0.1905 (CI: 0.03914 to 0.9334)) and were more likely to test positive than the rest of the population (p=0.018, OR=3.889 (CI 1.391 to 11.81)). Within this age group, there were nine patients, seven (77.8%) of whom had received an HCV test, with all testing positive.

## Discussion

The aim of this report was to evaluate the testing patterns and prevalence of HIV and HCV among patients presenting to the emergency department following an opioid overdose to assess the potential utility of implementing screening testing for these infections. Of the 120 patients included in this analysis who presented to the emergency department following an opioid overdose, 54 (45%) had a documented HIV test, while 48 (40%) had a documented HCV test, indicating that there is a paucity of testing for both infections in this population. Among those who had received testing, only one (0.8%) patient had a positive HIV test, while 20 (16.7%) patients had previously received a positive HCV test. This indicates that there was a high prevalence of HCV, justifying the implementation of universal opt-out screening for patients in the emergency department being held for observation following an overdose. With the low prevalence of HIV in this sample, there is insufficient evidence to establish a potential benefit for a similar screening program for HIV, though there is consensus in support of HIV screening in this population.

Overall HCV prevalence

Of the 48 patients who had ever received an HCV test, 20 had received a positive test. This indicated a minimum prevalence among the sample population of 16.7% (n=20) and a prevalence of 41.7% (n=20) among those who had ever received a test. Six (30%) of the 20 patients who had ever received a positive HCV test had a documented detectable viral load, giving a minimum prevalence within the study population of 5%. With the estimated prevalence of viremic HCV infections in the United States in 2020 being 0.8% [9], this study population had a markedly higher burden of HCV infection compared to the general population. With the estimated HCV seropositivity among people who inject drugs being 53.3% [10], there is likely a potential benefit for increased surveillance and linkage to care among this patient population.

Gender differences in patient population and HCV status

Males predominated the study population, comprising 72 (60%) of the 120 patients. This is consistent with previous estimates that in North America, women comprise 30% of people who inject drugs [10], a population that has long been recognized as having an elevated risk of HCV. In addition to presenting to the emergency department more frequently, males had testing results that were of particular concern. A total of 16.7% (n=12) of all male patients and 16.7% (n=8) of all female patients had tested positive for HCV. However, males were far more likely to have never received an HCV test, as the percentage of males who had never been tested for HCV in this sample was 23.6% higher than the percentage of females who had never been tested for HCV (n=72 and 60% for males vs. n=22 and 45.8% for females). Given this disparity, it is possible that the actual prevalence of HCV among males in this sample was higher than it was for women. This would be consistent with previous studies that have found that individuals infected with HCV are more likely to be male [11] and estimate the HCV seroprevalence in men to be twice the seroprevalence in women [12].

Age differences in HCV rates

The HCV positivity status was influenced by the age of the patients. Seven of nine (77.8%) patients aged 55-64 had received some form of HCV testing, and all had a positive test result, making this age group more likely to have ever received an HCV test and more likely to test positive for HCV. Each of these patients was born between 1945 and 1965. This birth cohort has an HCV prevalence that is estimated to be three times greater than that of the United States population and is estimated to account for 76.5% of all individuals with HCV antibodies [13]. Given the higher positivity rates among this age group, the use of opioids may amplify the risk of transmitting HCV to a greater degree among persons born 1945-1965 compared to other age groups, and thus particular attention should be dedicated to screening individuals from this birth cohort who use opioids.

HIV testing history and prevalence

Only 54 (45%) of the 120 patients in this review had ever received an HIV test. However, there was not a similarly high prevalence of HIV among those with testing, with only one patient (0.8%) having a documented positive HIV test. While the prevalence of HIV may be low within this sample, with greater than half of all individuals being untested, there may be a risk for a large HIV outbreak in central Missouri. A recent review of eight HIV outbreaks across Europe, North America, and Israel found that each community had a low prevalence of HIV prior to the outbreak. In many of these communities, this led to an attitude of complacency characterized by low surveillance and decreasing investment in harm-reduction programs [4]. With a low testing history among this high-risk patient population, central Missouri appears to share some of the same characteristics that predisposed other communities to HIV outbreaks.

Drug administration patterns

This study failed to find any association between the route of drug use and HCV testing history. This is likely due to the methods by which data regarding drug use was collected. Information related to the route of drug administration was based on narrative reports within emergency medical services transport documents and emergency department encounter notes. These sources relied primarily on self-reported data to determine the method by which patients used opioids. Given that these patients are often unable to provide a history following an overdose, this information is often unavailable. Additionally, fears regarding the perceived legal ramifications of drug use may have influenced the honesty of patients’ responses regarding their drug use.

Limitations

This study had multiple limitations. As with all observational studies, this study can only show associations between our measures of interest and cannot establish causality. As it is a retrospective chart review, there is the potential for incomplete documentation and variance in documentation patterns amongst different providers, potentially impacting the reliability of the results obtained. The modest sample size of this study resulted in only a small number of patients in the study who had positive tests for HIV and HCV, thus limiting the power of this study and the ability to draw definitive conclusions about implications for screening for these infections. In addition, this study was limited to health encounters in the emergency departments in only one health system. As all patients included in this study presented to the emergency department, the study population may differ from overdose patients who either did not receive emergency medical treatment or refused transport to the emergency department. Therefore, they may represent a subset of individuals who are more likely to utilize healthcare services and have different rates of testing for these infections. As a result, this population may not reflect the entire population of Missouri or the United States. Lastly, our electronic health record did not differentiate where HIV and HCV testing occurred, limiting the ability to comment on whether testing for these infections in the emergency department has been effective in identifying these infections to date.

## Conclusions

Patients presenting to University of Missouri emergency departments following opioid overdoses have high rates of HCV and there is a potential benefit for increased screening efforts in this population. In particular, individuals born between 1945 and 1965 have high rates of HCV, though they have higher rates of testing than any other age cohort. Men also show lower rates of testing and may have higher rates of HCV than women. There may be potential benefits in the implementation of universal opt-out testing for HCV for patients being held for observation following an opioid overdose.

The prevalence of HIV was low within this population, and there is insufficient evidence to state that implementation of universal opt-out testing for HIV following an opioid overdose in this population would lead to additional cases being identified. However, there is a similar lack of HIV testing within this population, potentially predisposing this community to a large HIV outbreak due to the unknown disease status of many individuals.
